# Association of circulating tumor cell-white blood cell clusters with survival outcomes in patients with colorectal cancer after curative intent surgery

**DOI:** 10.1186/s12876-022-02603-4

**Published:** 2022-12-06

**Authors:** Yifan Xu, Qianlong Zhang, Zhou Xu, Qingfeng Xie, Wenfu Ding, Hao Liu, Haijun Deng

**Affiliations:** 1 Department of General Surgery, Nanfang Hospital, Southern Medical University, 1838 North Guangzhou Ave, Guangzhou, 510515 China; 2 Ministry of Education-Shanghai Key Laboratory of Children’s Environmental Health, Xinhua Hospital, Shanghai Jiao Tong University School of Medicine, Shanghai, 200092 China

**Keywords:** CTC-WBC clusters, Circulating tumor cells, Neutrophils, Prognosis, Therapeutics

## Abstract

**Background:**

The analysis of circulating tumor cell-associated white blood cell (CTC-WBC) clusters represented the progress in the liquid biopsy of malignant tumors, however, related research in patients with colorectal cancer is still absent.

**Methods:**

To explore associations between CTC-WBC clusters and the prognosis of these patients, we conducted an independent cohort of 329 colorectal cancer patients after curative intent surgery and pre-operative CTC detection in Nanfang Hospital, Southern Medical University, Guangzhou, China between January 1, 2017, and September 31, 2019. The primary cohort referred to patients with CTC-WBC clusters positive. The control cohort was defined as those with exclusively CTCs positive. CTCs were enriched and distinguished by The CanPatrol™ system (SurExam, China). The Kaplan–Meier curve was used to compare the progressive-free survival (PFS) and overall survival (OS) between two groups. The COX regression model was used to assess the predictive value of CTC-WBC clusters.

**Results:**

Sixty three patients presented CTC-WBC clusters positive (CTC-WBC group) and 266 patients showed solely CTCs (CTC group). The number of CTCs was significantly different between two groups (*P* < 0.001) and the rest of clinical characteristics were not markedly associated with the presence of CTC-WBC clusters. Kaplan–Meier curves of PFS and OS exhibited that the CTC-WBC group had significantly shorter PFS (*P* = 0.011), while not for OS. The multivariate model further suggested that the CTC-WBC clusters (Hazard Ratio = 1.89, 95% Confidence Interval 1.02–3.51, *P* = 0.042) was an independent predictor for the PFS of in post-operation CRC patients.

**Conclusion:**

The CTC-WBC cluster is significantly associated with recurrence after operation in CRC patients. This finding facilitates the evaluation of this indicator in tumor progression.

## Background

Colorectal cancer (CRC) has the third-highest rate of estimated new cases and mortality worldwide [1]. The metastasis is found in 20% firstly-diagnosed CRC patients and among others who have only local lesions, 25% will develop metastatic clony(ies) in few months or years [2]. The intransigent metastasis and local recurrence are responsible for the majority of deaths for CRC patients. Common serological marker detection such as tumor cancer embryo antigen (CEA) and carbohydrate antigen 199 (CA199) have limited accuracy for CRC. Therefore, finding a sensitive and convenient avenue of the early discrimination for patients at a high risk of recurrence and metastasis has become an important issue for clinicians.

The theories concerning tumor metastasis are complex and diverse, one is that circulating tumor cells may travel as individual cells or clusters from the primary site to the microvascular of distant tissues [3]. The circulating tumor cells (CTC) have been detected in a penal of common carcinomas, such as breast [4], prostate [5], lung [6], renal [7], bladder [8], liver [9] colon [10] and gastric cancer [11]. Known as liquid biopsy, CTCs are easy to collect, reducing the burden of invasive tissue biopsies, and are enable to reveal overall cancer genome features instead of constrained information from isolated tumor samples [12]. Current efforts mainly focus on the quantitative abundance, biological characteristics and genomic heterogeneity of CTCs to evaluate the haematological metastasis, recurrence of tumors and the response of chemotherapy. Clinical decisions can be made as early as possible for those who represent poor prognosis or have resistance to cancer targeted drugs due to heterogeneity between the origin and metastatic lesion. Our previous study provided proof that CTCs exhibiting epithelial-mesenchymal transition (EMT) feature could serve as the monitor of therapy response in patients with gastric cancer before and after surgery as well as during adjuvant chemotherapy [13].

However, it is rather a challenge for tumor cells from the local site to overcome the high pressure and turbulences during blood borne dissemination, as well as the immune attack to seed in distant parenchyma, which makes it difficult for the detection of CTCs in a constant cut-off value [11]. Recent studies illustrated the concept that the migration ability of CTCs could be ameliorated by gathering into clusters, and further enhanced by close interaction of with white blood cells (WBC) and platelets in the blood microenvironment [14]. Szczerba et al. [15] has revealed the role of neutrophils as escorts in the blood steam to promote the cell cycle progression and accelerate metastasis formation in the breast cancer model. Researches seeking the crosstalk between the tumor prognosis and CTC-WBC clusters have been conducted in several cancers, including those of breast [15], lung [16], renal [17], liver [18] and gastric [19], but absent for CRC.

To investigate the prognostic value of CTC-WBC clusters in CRC, we conducted a retrospective, single-center study, comparing the progression free survival (PFS) and overall survival (OS) between the CTC-WBC clusters positive group and the exclusively CTCs positive group in post-operative CRC patients based on a prospectively registered database in China.

## Methods

### Study design and patients

For this retrospective cohort study, we included an independent cohort of 329 CRC patients in total who had received palliative or radical surgery and CTC detection in Nanfang Hospital, Southern Medical University, Guangzhou, China. Patients were included between January 1, 2017, and September 31, 2019. The blood was drawn from patients for CTC testing approximately 1 week before they received surgery in our hospital and the primary data were collected in the database for later analysis. We constructed the primary cohort with patients who had a confirmed detection of CTC-WBC clusters positive. The control cohort was defined as the exclusively CTCs positive. The study was approved by the ethics review committee of Nanfang Hospital, Southern Medical University, Guangzhou, China. None of the patients were treated with steroids or anti-inflammatory drugs that could affect blood immune cell counts before blood sample collection.

### Data collection

The baseline data were obtained from the database of our hospital’s electronic medical record system, including gender, age, histological type, surgery type, depth of invasion, lymph node metastasis, postoperative liver metastasis etc. Blood detection biomarkers included CTC data (number of CTCs, type of CTCs, CTC-WBC clusters) and pre-operative CEA, CA199. The disease progression was obtained from our follow-up system, including the disease condition after surgery, the date of recurrence, metastatic or death. Tumor staging was based on the 8th edition of the American Joint Committee on Cancer (AJCC) staging manual [20]. OS was calculated from the date of surgery until death due to any cause. PFS covered the period from the date of surgery to disease progression or death occurred due to any reasons. The last follow-up date was August, 2020. We choose CTC data from 2017 to 2019 to explore associations between CTC-WBC clusters and the prognosis of CRC patients retrospectively.

### Isolation and enumeration of CTCs and CTC-WBC clusters

**Fig. 1:**
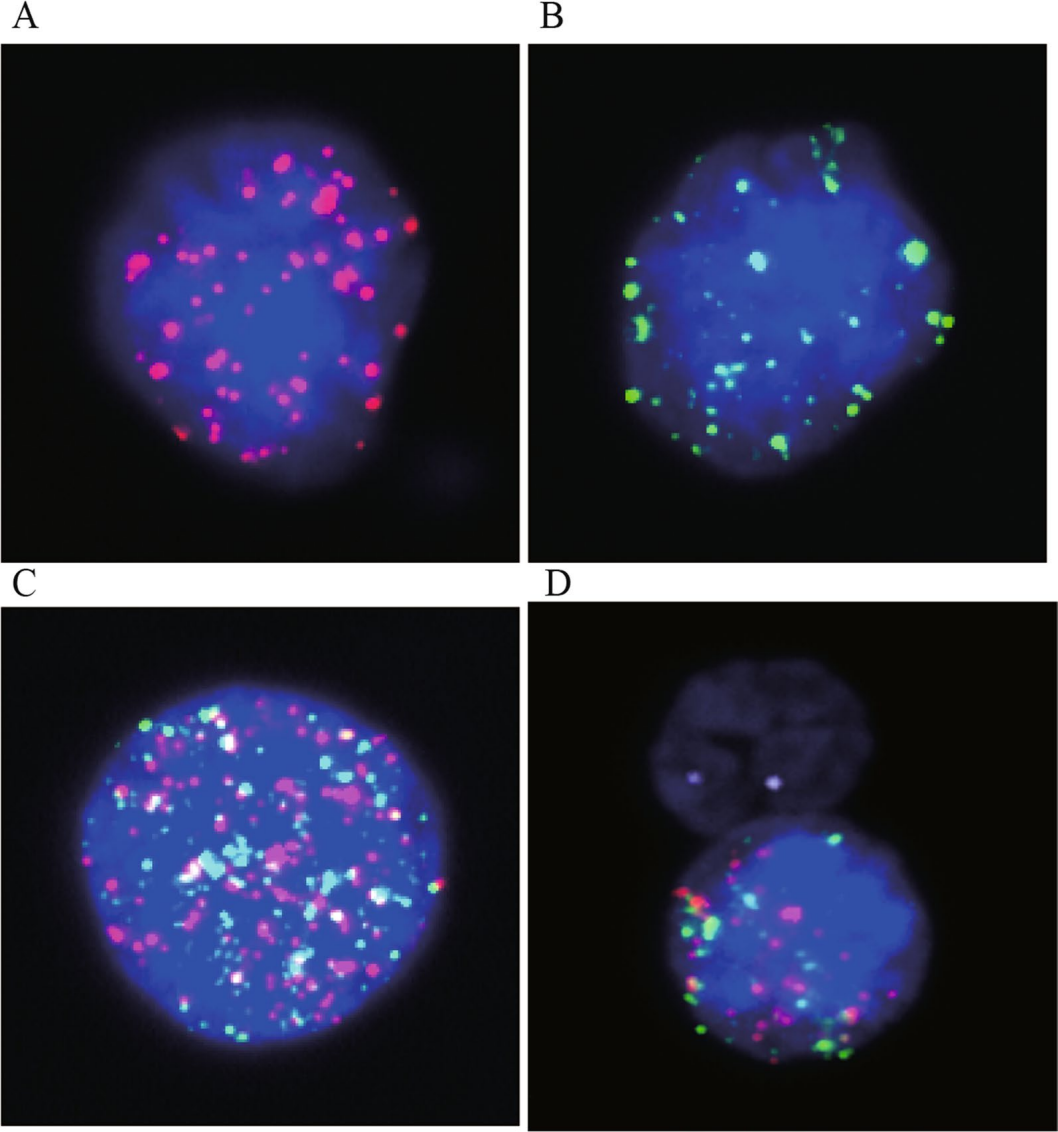
The epithelial type presented as the red fluorescent signal points (**A**); the mesenchymal type presented as the green fluorescent signal points (**B**); the mixed type presented as both red and green fluorescent signal points (**C**); CTC-WBC cells appeared as white dots surrounded by red, green, or red/green fluorescent signals points (**D**)

5 ml peripheral blood samples were collected from CRC patients before surgery in EDTA tubes. CTCs were enriched and distinguished into subgroups by the CanPatrol™ CTC analysis system (SurExam, China) within 72 h after intravenous blood drawn. The collecting process was based on nano membrane filtration. The ribonucleic acid in situ hybridization (RNA-ISH) method was adopted to subtype CTCs. The epithelial type was identified using fluorescently labeled antibodies against the epithelial cell adhesion antigen (EpCAM), cytokeratoin 8, 18, and 19. The mesenchymal type was detected by the expression of Vimentin and Twist protein. Leukocyte biomarkers CD45 were used to intercept CTC-WBC clusters. The red fluorescent signal dots represented epithelial-like CTCs and the green dots referred to mesenchymal-like CTCs. The mixed type was observed as a hybrid of red and green dots. CTC-WBC clusters labelled by CD45 were stained as white dots scattered by red, green, or red/green mixture under the automated fluorescence microscope (Fig. 1).

### Statistical analysis

All data were collected and analyzed in SPSS version 22.0 (IBM) and R version 4.0.2. The histological type of CRC was classified into nonspecific adenocarcinoma, specific adenocarcinoma (mucosal adenocarcinoma, signet ring cell carcinoma) and the mixed. The threshold of CTC number was set according to the median. The baseline data were assessed with the unpaired, 2-tailed Pearson's chi-square tests after the patients were divided into cohorts. The Kaplan–Meier curve was used to compare the prognosis for the CTC-WBC positive group and the exclusively CTC positive group. The Kaplan–Meier curve was checked by the log-rank test. We used the univariate cox proportional hazards analysis to assess the predictive value of CTC-WBC clusters and other indicators. Relevant influencing factors were incorporated in the multivariate cox proportional hazards analysis to compare PFS. In general, 2-sided *P* < 0.05 indicates a statistically significant difference.

## Results

### Baseline characteristics

In this study, a total of 329 post-operation CRC patients were consecutively included. All of them were evaluable and admitted in the final analysis. Among these patients, 218 (66.3%) were men and 111 (33.7%) were women. The age ranged from 16 to 88 and the median age was 58. Most patients have undergone radical surgery (291 cases, 88.4%), the remaining patients were diagnosed as stage IV and could only receive palliative surgery (38 cases, 11.6%). The disease severity was staged according to the TNM system, where 41 (12.5%) patients with stage I, 114 (34.7%) patients with stage II, 144 (43.8%) patients with stage III and 30 (9.1%) patients with stage IV. The median number (Inter-quartile Range) of CTCs was 6/5 mL blood (1–116 mL blood), and 176 (53.5%) patients showed the number of CTCs >6. For the type of CTCs, mesenchymal phenotype was expressed in 296 (90.0%) patients. The clinicopathological features classified by CTC-WBC clusters are summarized in Table 1.

**Table 1:** Clinicopathological features in postoperative colorectal cancer patients

Features	Group, n (%)		*P*-value
	CTC-WBC	CTC	
Age (years)			0.457
≥ 65	19 (30.2)	68 (25.6)	
< 65	44 (69.8)	198 (74.4)	
Gender			0.335
Male	45 (71.4)	173 (65.0)	
Female	18 (28.6)	93 (35.0)	
Type of surgery			0.45
Radical surgery	54 (85.7)	237 (89.1)	
Palliative surgery	9 (14.3)	29 (10.9)	
Histological type			0.835
Nonspecific adenocarcinoma	58 (92.1)	236 (88.7)	
Specific adenocarcinoma	2 (3.2)	15 (5.6)	
Mixed	3 (4.8)	15 (5.6)	
Depth of tumor invasion			0.096
pT4	23 (36.5)	132 (49.6)	
pT3	29 (46.0)	91 (34.2)	
pT2	10 (15.9)	29 (10.9)	
pT1	1 (1.6)	14 (5.3)	
Lymph node metastasis			0.468
pN2	17 (27.0)	53 (19.9)	
pN1	17 (27.0)	78 (29.3)	
pN0	29 (46.0)	135 (50.8)	
pTNM stage			0.383
IV	9 (14.3)	21 (7.9)	
III	26 (41.3)	118 (44.4)	
II	19 (30.2)	95 (35.7)	
I	9 (14.3)	32 (12.0)	
Postoperative liver metastasis			0.274
Liver metastasis	5 (7.9)	10 (3.8)	
Non-liver metastasis	58 (92.1)	256 (96.2)	
Preoperative CA199			0.175
≤ 37, ug/L	52 (82.5)	199 (74.8)	
> 37, ug/L	10 (15.9)	46 (17.3)	
Missing	1 (1.6)	21 (7.9)	
Preoperative CEA			0.296
≤ 5, ug/L	45 (71.4)	168 (63.2)	
> 5, ug/L	16 (25.4)	76 (28.6)	
Missing	2 (3.2)	22 (8.3)	
Number of CTC			< 0.001
≤ 6	21 (33.3)	155 (58.3)	
> 6	42 (66.7)	111 (41.7)	
Type of CTC			0.433
Mesenchymal or mixed	55 (87.3)	241 (90.6)	
Epithelial only	8 (12.7)	25 (9.4)	

### Comparisons of CTC versus CTC-WBC

Among 329 patients, 63 (19.1%) patients presented CTC-WBC clusters positive (CTC-WBC group) and 266 (80.9%) patients showed solely CTCs (CTC group). The number of CTCs, which divided by the median 6 was significantly different between two groups (*P* < 0.001). We aimed to compare the influence of CTC-WBC clusters on liver metastasis after the surgery, while there is no significant difference between the two (*P* = 0.274). Next, we investigated whether immune cells induce epithelial-mesenchymal transition feature. Although only 33 (10.0%) patients showed exclusively epithelial CTCs, the CTC-WBC cluster was not correlated with EMT (*P* = 0.433). The rest of clinical characteristics was not markedly associated with the present of CTC-WBC clusters.

### Survival outcomes

**Fig. 2:**
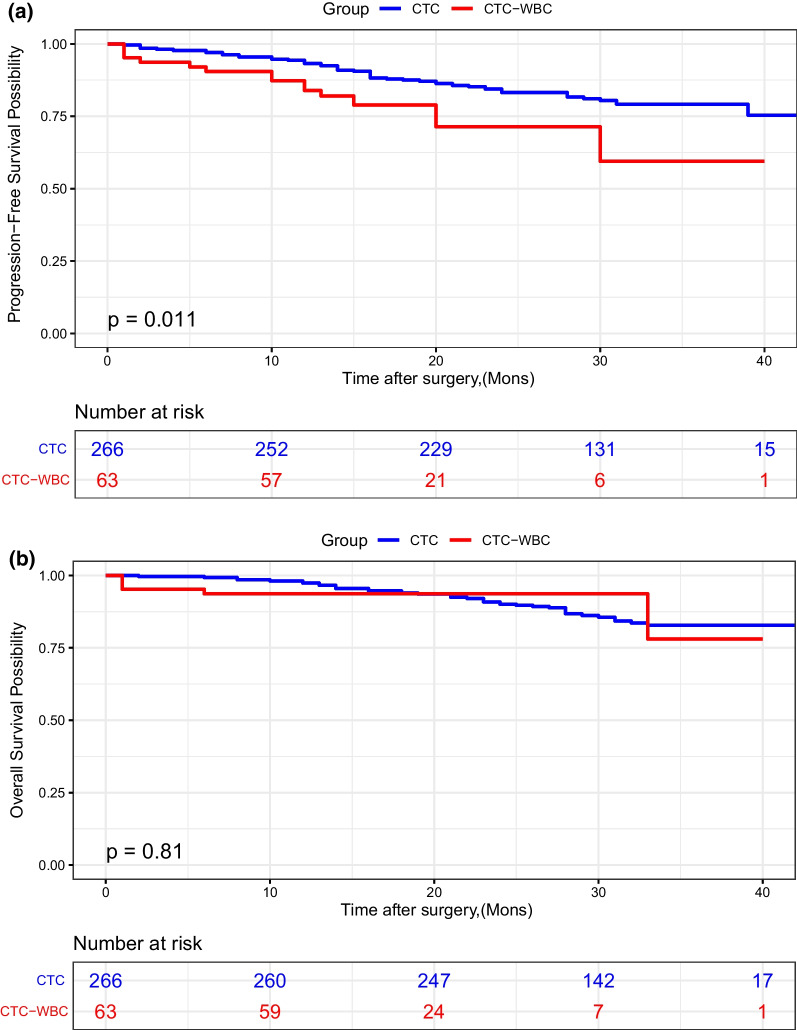
Correlation of CTC group and CTC-WBC group with PFS (**a**) and OS (**b**) in post-operation CRC patients

The follow-up duration ranged from 11 to 43 months, with a median of 30 months. Eighteen patients have received pre-operative neoadjuvant chemotherapy, among them 2 patients showed pathological complete response, 1 showed stable disease, the others showed partial response. There were 44 (13.4%) deaths in total and three of them died of non-neoplastic causes. Kaplan–Meier curves of PFS and OS were illustrated to compare the CTC-WBC and CTC group, exhibiting that the CTC-WBC group showed significantly shorter PFS (*P* = 0.011, Fig. 2A) than the latter, while not for OS (*P* = 0.72, Fig. 2B). The univariate Cox risk proportional regression analysis demonstrated that the surgery type [Hazard Ratio (HR) = 6.18, 95% confidence interval (CI) 3.74–10.22, *P* < 0.001], tumor stage (HR = 5.36, 95% CI 2.81–10.23, *P* < 0.001), preoperative CEA (HR = 2.11, 95% CI 1.28–3.50, *P* = 0.004), and CTC-WBC/CTC group (HR = 2.10, 95% CI 1.17–3.79, *P* = 0.013) were significantly correlated with the progression free survival (Table 2). The multivariate model further suggested that the CTC-WBC cluster (HR = 1.89, 95% CI 1.02–3.51, *P* = 0.042), the surgery type (HR = 3.52, 95% CI 1.98–6.24, *P* < 0.001) and the pTNM stage (HR = 3.39, 95% CI 1.69–6.80, *P* = 0.001) were all independent predictors for the PFS of post-operative CRC patients. (Table 2).

**Table 2:** Univariate and multivariate analyses of PFS in post-operation CRC patients

Independent factor (DFS)	Univariate analysis		Multivariate analysis	
	Hazard ratio (95%CI)	*P*-value	Hazard ratio (95%CI)	*P*-value
Age (< 65/≥ 65, years)	0.97 (0.56–1.69)	0.899		
Gender (male/female)	1.01 (0.61–1.69)	0.961		
Type of surgery (palliative surgery/radical surgery)	6.18 (3.74–10.22)	< 0.001	3.52 (1.98–6.24)	< 0.001
Histological type (nonspecific adenocarcinoma/specific adenocarcinoma or mixed types)	0.56 (0.29–1.06)	0.075		
pTNM stage (III–IV/I–II)	5.36 (2.81–10.23)	< 0.001	3.39 (1.69–6.80)	0.001
Preoperative CA199 (> 37/≤ 37, ug/L)	1.29 (0.71–2.35)	0.401		
Preoperative CEA (> 5/≤ 5, ug/L)	2.11 (1.28–3.50)	0.004	1.25 (0.74–2.13)	0.407
Number of CTC (> 6/≤ 6)	1.35 (0.83–2.18)	0.224		
Group (CTC-WBC/CTC)	2.10 (1.17–3.79)	0.013	1.89 (1.02–3.51)	0.042

## Discussions

In the present study, we found that CTC-WBC cluster was associated with worse progressive-free survival in patients with colorectal cancer after radical or palliative surgery. Furthermore, the multivariate analysis underscored the independent influence of CTC-WBC clusters for PFS as well. The detection of changing levels of CTCs provides a “liquid biopsy” approach to monitor therapy responses during the course of treatment. Compared to medical image scans and laparoscopy, CTC detection commonly costs less and is conducted more conveniently with a blood test. It could give clues for tumor progression before measurable changes found in the image, as early as 4 weeks from the beginning of therapy [21]. As for routine serological examination like tumor markers, researches show that the surveillance of CTC have an increased sensitivity without the disturbance from nonmalignant conditions [22]. From the count of single CTC, to the comprehensive acquirement of tumor functional representatives, this weapon has been continuously optimized with multiple detection strategies including a package with WBCs. Our findings hold promise for the CTC-WBC cluster becoming a new therapeutic target through disassembling of this package.

Long before the last decade, the value of CTC for tumor prognosis had been understood by oncology researchers, and a large number of studies using cytometric methods or polymerase chain reaction (PCR)-based approaches had been conducted in colorectal cancer and confirmed the prognostic value of CTC. Later, the CellSearch system (Veridex LLC) was established and became popular [23, 24]. However, this method using anti-EpCAM-coated magnetic beads to capture markers absent from leukocytes has considerable limitations in detecting EMT CTCs, which have been proven to be linked to a worse treatment response in gastric cancer patients by our previous study [13]. In addition, due to the low detection rate of CTC in patients with early staged CRC, the uncertainty of the CTC cut-off value will bewilder the interpretation of the carcinoma prediction by CTC. In a prospective multicenter study of 519 patients with stage III CRC recruited, no significant association was seen between CTC enumeration and the prognosis [25]. Nevertheless, there has been an increased interest regarding the possible use of the association between tumor cells and immune cells. In the present study, we used an improved CanPatrol™ CTC enrichment technique to unbiasedly isolate CTCs and CTC-WBC clusters. The system combined the superiority of CD45+ leukocytes depletion and isolation of CTCs by size, which is more efficient and duplicable for CTC gathering and enumeration [26].

The role of tumor-associated neutrophils (TANs) as a driver of tumor progression have been drilled down deeply by previous studies. Specific examples of transforming growth factor (TGF)-β shaping the pro-tumorigenic (N2) or antitumorigenic (N1) effects of TAN were demonstrated [27]. However, trials exploring the correlation between CTC-WBC clusters and the prognosis in cancer patients have been blank until the inspiration by Szczerba et al. [15]. The research revealed the mechanism of interactions between CTCs and WBC-clusters in breast cancer mouse models. They found that the proportion of neutrophils in CTC-WBC clusters ranged from 85.5 to 91.7% whereas only a minority (8.3–14.5%) were monocytes, which was consistent with studies reporting an association between a higher neutrophil-to-lymphocyte ratio (NLR) and poor clinical outcomes in various cancers [28]. Szczerba et al. also showed that CTCs from CTC–WBC clusters were predisposed to be exuberant in positive regulators of cell cycle and deoxyribonucleic acid (DNA) replication programs compared to CTCs alone and could express genes that encode granulocyte colony-stimulating factor (G-CSF), which facilitated pro-tumor activities like neutrophil recruitment, Bv8 expression and angiogenesis [29–31]. On the contrary, genes involved in EMT were not related with the interaction between CTCs and neutrophils. Accordingly, in our study, although only 33 (10%) patients expressed epithelial solely CTC, no significant association was observed between CTC-WBC positive and mesenchymal phenotype. However, the sample size derived from patients in Szczerba’s study were small with merely 34 heterogenous CTC-detectable patients. In the absence of confounding cause adjustment, the bonus influence of CTC-WBC clusters could be attenuated.

Although TANs were identified as the supporting factors mediating distant CRC metastasis, especially liver metastasis [32], the therapeutic benefit of CTC-WBC clusters in CRC patients has not been studied so far. Our study mainly focused on validating the prediction value of CTC-WBC clusters in CRC patients after surgery. By means of the Kaplan–Meier survival curves, a significant shorter progression-free survival period could be seen compared with the CTC-WBC negative group. The univariate Cox risk proportional regression analysis proposed that the surgery type, tumor stage, preoperative CEA and CTC-WBC/CTC group were all independent predicting factors correlated with poor PFS and the multivariate analysis further suggested that CTC-WBC clusters was an independent predictor for the PFS of post-operative CRC patients after adjustments with all of these factors.

The study has several limitations. The significant association with CTC-WBC clusters was neither exhibited in the overall survival, nor the post-operative liver metastasis. Among the primary culprit for causing is the small number of cases from the single-center. Also, a longer follow-up and a consistent CTC monitor after the surgery are needed. The first two factors induce insufficient end-point events, while the latter indicates the lack of pre- and post-operative comparisons and subsequent long-term surveillance. In addition, the post-operative chemotherapy information of patients was not included due to the discharge of patients, which prevented us from adjusting the influence of adjuvant chemotherapy on prognosis or evaluating the therapy efficacy via CTC-WBC clusters. The failure in predicting liver metastasis may be explained by these reasons. Finally, the study was limited by the inherent flaws of retrospective studies and wanted in an extensive exploration of molecular mechanisms as we mentioned above. More work will need to be done to establish multi-center prospective studies with larger-scale samples and a longer follow-up duration.

## Conclusion

In summary, our study found the presence of CTC-WBC clusters in peripheral blood of preoperative CRC patients. Furthermore, we demonstrated that CTC-WBC cluster at baseline was significantly associated with worse progression-free survival and could serve as an independent predictor for CRC patients undergone radical or palliative surgery, while not for the overall survival. These clinical sample data provide clues for a more convenient and predictive monitoring for CRC patients and indicate a supplementary tool to early identify the occult progression of tumors. The prognosis could be improved by the timely adjustment of chemotherapy and surgery program. It is worth noting that larger-scale, multi-center and prospective studies are needed to obtain more convincing evidence.

## Data Availability

The datasets used and/or analysed during the current study are available from the corresponding author on reasonable request.

## Abbreviations

CRC: Colorectal cancer
CEA: Cancer embryo antigen
CA199: Carbohydrate antigen 199
CTC: Circulating tumor cell
EMT: Epithelial-mesenchymal transition
WBC: White blood cell
CTC-WBC: CTC-associated white blood cell
PFS: Progression free survival
OS: Overall survival
RNA-ISH: Ribonucleic acid in situ hybridization
EpCAM: Epithelial cell adhesion antigen
CI: Confidence interval
HR: Hazard ratio
PCR: Polymerase chain reaction
TAN: Tumor-associated neutrophils
TGF: Transforming growth factor
N1: Antitumorigenic
N2: Pro-tumorigenic
NLR: Neutrophil-to-lymphocyte ratio
G-CSF: Granulocyte colony-stimulating factor
DNA: Deoxyribonucleic acid
